# Detection of *Verticillium* species in Swedish soils using real-time PCR

**DOI:** 10.1007/s00203-017-1412-z

**Published:** 2017-07-24

**Authors:** Georgios Tzelepis, Sarosh Bejai, Muhammad Naeem Sattar, Arne Schwelm, Jonas Ilbäck, Johan Fogelqvist, Christina Dixelius

**Affiliations:** 1 Department of Plant Biology, Uppsala BioCenter, Linnean Center for Plant Biology, Swedish University of Agricultural Sciences, P.O. Box 7080, 75007 Uppsala, Sweden; 2 Present Address: Department of Plant Virology, Institute of Agricultural Sciences (IAGS), University of the Punjab, Quaid-e-Azam Campus, Box. 54590, Lahore, Pakistan; 3 Present Address: National Food Agency, Sweden, Box 622, 751 26 Uppsala, Sweden

**Keywords:** *Beta vulgaris*, *Brassica napus*, qPCR, Soilborne pathogens, *Verticillium*

## Abstract

*Verticillium* species are soilborne plant pathogens, responsible for big yield losses worldwide. Here, we report improved procedures to generate DNA from *Verticillium* species imbedded in farm soils. Using new genomic sequence information, primers for *V. dahliae*, *V. albo*-*atrum, V. tricorpus,* and *V. longisporum* were designed. In a survey of 429 samples from intensively farmed soil of two Swedish regions, only *V. dahliae* and *V. longisporum* were identified. A bias towards *V. longisporum* (40%) was seen in the south, whereas *V. dahliae* was more frequent in the western region (19%). Analyses of soil and leaf samples from 20 sugar beet fields, where foliar wilting had been observed, revealed *V. dahliae* DNA in all leaf and soil samples and *V. longisporum* in 18 soil samples, illustrating host choice and longevity of the *V. longisporum* microsclerotia. This study demonstrates the applicability of new molecular diagnostic tools that are important for growers of variable crops.

## Introduction

The angiosperm family *Brassicaceae* includes many economically important vegetables, condiment and fodder crops as well as edible and industrial oilseed (Rakow 2004). In terms of production, winter oilseed rape (*Brassica napus*) is the most important oil crop in Europe and globally third behind palm and soybean oil. Annual yields of winter oilseed rape in northern Europe are about one metric ton less compared to Central Europe (http://ec.europa.eu/eurostat), and the cause of this difference is debated. Besides slightly different climate and somewhat unpredictable climate conditions, one important factor for yield losses of *Brassica* crops in Sweden could be related to soilborne pathogens, such as *Verticillium longisporum* and *Plasmodiophora brassicae.* The latter causes the clubroot disease. *P. brassicae* was detected in more than 50% of analyzed soils from southern Swedish fields 2013 and 2014 and constitute an emergence disease threat to Brassica crop production (Wallenhammar et al. 2016).

*Verticillium dahliae* causes wilting disease on more than 200 different host plants (Pegg and Brady 2002), whereas *V. longisporum* is more restricted in its host range, attacking mainly plants in the *Brassicaceae* family. Although *V. longisporum* substantially shares the disease cycle characteristics with the more studied *V. dahliae,* it does not cause wilting of the plants. Therefore, the *V. longisporum* disease is now suggested to be named Verticillium stem stripe instead of Verticillium wilt (Depotter et al. 2016). A major problem for the disease management of these two plant pathogens is the longevity of their microsclerotia that are released into the soil from infected plant residues at the end of the disease cycle. These resting structures have the capacity to remain dormant in the soil for many years. It is anticipated that microsclerotia are stimulated to germinate by root exudates released from a host plant growing nearby. The hyphae of *V. longisporum* invade the lateral roots and root hairs (Zhou et al. 2006), colonizing the root tissues before enter the xylem elements. Here conidia are formed and can be spread via the plant transpiration stream. The colonization of the vessel tissue restricts the access to xylem sap of the plant cells and thereby affects the plant growth. Since microsclerotia are not formed and protruded in stems and leaves until the plant is in the senescence phase, a process associated with induction of abscisic acid (Roos et al. 2014), the infection commonly remains unnoticed.

Although not necessarily observed every year, the *Verticillium* stripe disease is more frequently reported in recent decades (Dixelius et al. 2005). Fluctuation in disease reporting is mostly explained by the late occurrence of disease symptoms in the season and a combination of new more tolerant crop varieties and impact of environmental factors influencing the microsclerotia germination frequency. However, early detection of *Verticillium* infested soils is crucial to prevent further spread and multiplication of these pathogens. Currently, detection methods used for *V. dahliae* or *V. longisporum* are either based on soil sieving and plating on agar plates followed by counting of germinating microsclerotia (e.g. Goud and Termorshuizen 2003; Kabir et al. 2004; Goud et al. 2011) or deployment of quantitative polymerase chain reaction (qPCR). Plate methods are laborious and require expertise when monitoring the colonies on the plates, whereas qPCR is a fast, unbiased and a sensitive method to detect and quantify individual species. However, qPCR relies on the use of proper primers and reasonable pure DNA template and fails to discriminate between DNA from viable and dead fungal structures.

This work had the overall aim to lay the grounds for a reliable, sensitive and high-throughput molecular detection method of natural *Verticillium* infested soils. This knowledge could give the ability to growers to test the level of soil infestation prior to sowing and to optimize their crop rotation schemes.

## Materials and methods

### Fungal isolates, cultures and DNA extraction

DNA from single-spore isolates of four *Verticillium* species (Table 1) was used as reference materials to test the specificity of the primers design. All individual fungal isolates were grown on potato dextrose agar (PDA) for two weeks and DNA was extracted from mycelium using a CTAB method (Moller et al. 1992). The DNA was quantified using a NanoDrop Micro Photometer. Microsclerotia of *V. dahliae* and *V. longisporum* were obtained from in vitro grown oilseed rape stems and further cultured as described by Neumann and Dobinson (2003). Finally, water suspensions were made by serially dilutions to achieve 25, 50, 500, 5000 and 10,000 microsclerotia per ml. One millilitre of these suspensions was used to artificially infest 500 g soils (Hasselfors, autoclaved and pre-analyzed for traces of *Verticillium*) followed by DNA extractions.

**Table 1:** *Verticillium* species and strains used as reference materials

*V. albo*-*atrum*	*V. longisporum*	*V. dahliae*	*V. tricorpus*
234	Bob-7	2341	2933
CBS-8	Vd-1	330	2901 A
CBS-7	43-3	G12-1	CBS-12
2934	Vd-11	Vd-7	1901
		G12-2	

Background data on the isolates can be found in Fahleson et al. (2003)

### DNA extractions from artificially infested soils

Soil samples were air dried at room temperature, milled and sieved through 106 and 20 µm nested sieves prior to any analysis. To avoid overloading, 100 mg soil powder was used when evaluating tested DNA extraction methods. DNA was extracted using (1) a bead-beating technique, (2) a freeze–thaw technique, (3) density flotation or (4) using commercial DNA kits. In the manual DNA extraction methods, soil was homogenized by vortexing in 1 ml extraction buffer (500 mM Tris–HCl pH 8.0, 100 mM sodium EDTA pH 8.0, 1.5 M NaCl). For the bead beating cell disruption (100 mg), samples were homogenized using the FastPrep^®^-24 (MPbio) with 100 mg of 0.1 mm diameter zirconia beads for 2 × 30 s at maximum speed 6 with intermittent storage on ice for 10 min. Freeze–thaw disruption of cells in the soil samples was achieved by three cycles of freezing in −70 °C overnight and thawing at 55 °C in a water bath for 15 min. Alternatively, a density flotation of the soil samples was carried out prior to the DNA extraction to further enhance yields. Here, 10 g soil was sedimented in 65% sucrose by centrifugation at 3000*g* for 15 min. The supernatant was passed through 0.2 μM filters (Millipore), the material retained on the filter was washed with sterile water and dissolved in water.

Samples obtained from either approach were incubated with the addition of proteinase K for 30 min at 37 °C, mixed with 20% (w/v) SDS and incubated at 65 °C for 1 h before centrifugation, at 4000*g* for 10 min. The supernatants were transferred to new tubes containing 0.5 volume of polyethylene glycol (PEG) solution [30% (w/v) PEG, in 1.5 M NaCl] and incubated at room temperature for 2 h prior to centrifugation. Pellets were dissolved in TE buffer (pH 8.0), mixed with 10 M NH_4_OAc and incubated on ice for 5 min. After centrifugation, the DNA in the supernatant was purified by phenol chloroform–isoamyl alcohol extraction, and isopropanol precipitation. The DNA was re-suspended in TE buffer and treated with RNAse before storage at −20 °C. DNA extraction using the commercial E.Z.N.A. soil DNA kit (Omega Bio-Tek), the ZR soil microbe DNA kit (Zymo Research) and the FastDNA spin kit (MP Biomedicals) for soil was performed with 350 mg soil according to the manufacturer’s instructions. DNA from leaf tissues was extracted using the GeneJET Plant Genomic purification kit (Thermo Fisher).

### PCR analysis

Genomic fungal DNA (5–10 ng) was used as template for PCR analysis using Phusion high-fidelity DNA polymerase (Thermo Fischer) in the following conditions: initial denaturation at 95 °C for 2 min followed by 30 cycles of 96 °C for 15 s, melting *T* +3 °C for 30 s, 72 °C for 35 s, followed by final extension of 72 °C for 5 min before storage.

### Real-time PCR

Real-time PCR assays were carried out using 5–10 ng total genomic DNA as template in a SYBR Green setup. All reactions were done at least as triplicates under following conditions: initial denaturation at 95 °C for 10 min followed by 30–40 cycles of 96 °C for 15 s, 60 °C for 30 s, 72 °C for 30 s, using the CFX Connect Real-Time System (Bio-Rad). The same protocol was used for all the primers tested. A melting curve analysis was done at the end of each run to verify the presence or absence of target sequences and to distinguish specific amplifications from nonspecific ones and primer dimers.

### Amplification inhibition

Any potential qPCR inhibition was monitored by comparing the internal positive control Ct value obtained with soil extracts to those obtained with nuclease-free water samples (negative control). DNA extracted with the conventional methods described above, required 200-fold dilution to overcome inhibition or reactions in the amplification step. For the three commercial kit extracts, a tenfold dilution was enough to eliminate any traces of inhibition in the qPCR. The soil samples spiked with various amounts of microsclerotia from *V. dahliae* and *V. longisporum* used for the DNA extractions were also used to determine the sensitivity of the qPCR analysis.

### DNA quantification

Purified DNA, isolated from the *Verticillium* strains, was quantified using Qubit (Invitrogen). The standard curve technique was applied with a tenfold dilution series of DNA from each *Verticillium* species in sterile water ranging from 100 to 1 × 10^6^ fg DNA/5 μl. The slope of the standard curve was used to determine the PCR efficiency (E = 10^−1/slope^ − 1). For quantification of *Verticillium* species in DNA from sugar beet leaves, primers of the endogenous adenylate transporter gene developed by Chaouachi et al. (2013) were used. All calculations and statistical analyses were performed as described in the ABI PRISM 7700 Sequence Detection System User Bulletin #2 (Applied Biosystems, USA) as outlined by Martin et al. (2011).

### Naturally infested field samples

Soil samples from southern and western regions in Sweden were collected during springtime of 2013, 2014 and 2015. Each soil sample consisted of 10 subsamples taken to a depth of 20 cm in a W-pattern from each field. The samples were homogenized, air dried at room temperature, milled and sieved (Wallenhammar et al. 2012). Three hundred fifty mg soil powder was used in all analysis of field materials, the quantity used by commercial companies and DNA was extracted using the FastDNA spin kit (MP Biomedicals). In addition, samples of *Verticillium* infected leaves and soil from 20 sugar beet fields from the southern region were collected and analyzed.

## Results and discussion

### DNA preparation from artificially infested soil

Soil is a complex substrate for DNA extraction. Factors such as texture, pH and organic matter, including humic substances, impact quality and quantity of DNA and may influence downstream molecular analysis (Schena et al. 2013). To improve this part of the analysis, we first used samples with known amount of microsclerotia mixed in greenhouse soil. Pretreatments such as bead-beating and freeze–thaw methods did not yield in any significant increase in the DNA amounts compared to density floatation. The latter method was a less complex density floatation procedure compared to Debode et al. (2011). Our protocol yielded about 2.6 times higher amount of DNA compared to the other two methods, but the procedure is still more labor intense than the bead-beating and freeze–thaw methods.

Three commercial kits were also included in the comparisons. In our conditions, the FastDNA SPIN kit yielded the highest average amount of fungal DNA per gram of soil compared to all other methods and kits used. The lowest detection level obtained from the artificially infested soils (25 microsclerotia) was 63 fg DNA of *V. dahliae* and 100 fg DNA for *V. longisporum.*

### Primer design and specificity assays

Earlier published qPCR primers were first tested on DNA of our reference strains. Those primers showed either reduced amplification efficiency (Atallah et al. 2007), formation of primer dimers (Pantou et al. 2006; Atallah et al. 2007; Lievens et al. 2006), or absence of species specificity (Pantou et al. 2006; Eynck et al. 2007; Atallah et al. 2007; Inderbitzin et al. 2011, 2013).

**Fig. 1:**
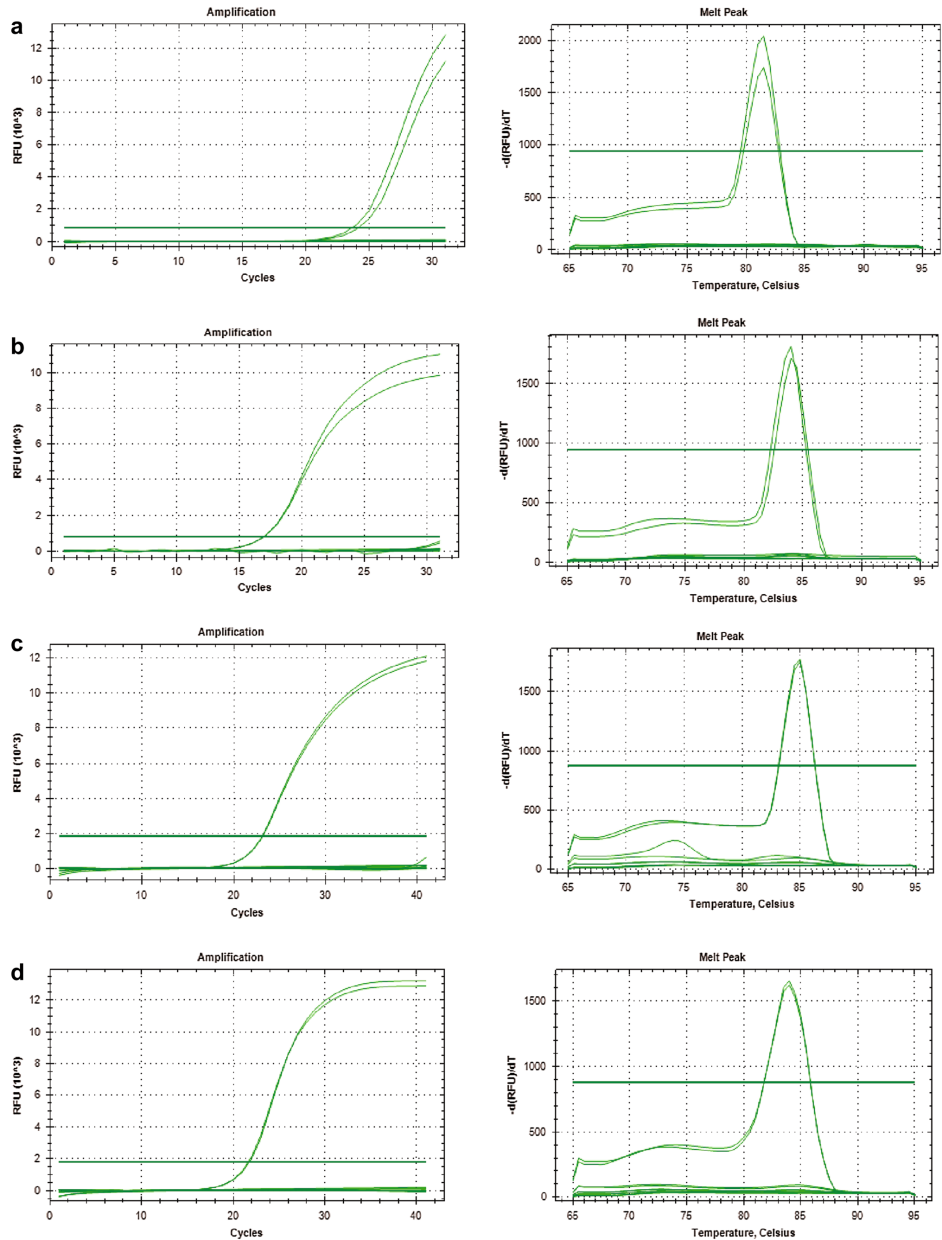
Analysis of *Verticillium* primers specificity. Primers listed in Table 2 of **a** *V. longisporum*, **b** *V. dahliae*, **c** *V. albo*-*atrum*, **d** *V. tricorpus* were tested against the other *Verticillium* species. Amplification was observed only on-target *Verticillium* species. No amplification on off-target species and on mock samples was observed. Melt curve analysis shows amplification of a single fragment. Two technical replicates were used

Universal species-specific primers for four *Verticillium* species (*V. dahliae, V. albo*-*atrum. V. tricorpus, V. longisporum*) were designed, by comparing the genome sequences of *V. dahliae* and *V. albo*-*atrum* (Klosterman et al. 2011) and the genome assembly data of two in house-generated Swedish *V. longisporum* isolates (unpublished data). Sequences were aligned and searched for single nucleotide polymorphisms (SNPs) and species-specific regions. Twenty-four primer pairs were designed in regions potentially unique to each species. Specificity of each primer pairs was tested by PCR and qPCR analysis using DNA extracted from isolates of the four species (Table 1). To test for any cross-reactivity, mixtures of DNA from one *Verticillium* species, and from another *Verticillium* strain were tested followed by qPCR analysis with primers for each individual species. All information together with melting curve analysis and the lowest Ct values, resulting in a single fragment, was the criteria set to identify the most optimal primer pair for each species (Fig. 1; Table 2). Specificity for primers enlisted in Table 2 was further checked against different soilborne pathogens such as *Rhizoctonia solani*, *Phytophthora pisi* and *Aphanomyces euteiches*. No amplification was observed when DNA from any of these species was used in the amplification reactions (data not shown).

**Table 2:** List of *Verticillium* species-specific primers used in qPCR analysis

Species	Forward 5′–3′	Reverse 5′–3′
*V. longisporum*	CGAGGAGTGAAAAGAAAACGGTTA	CGCGCCGAGGCTAGTCAC
*V. dahliae*	TCCTAGGCAGGCGAGCAG	TAGGGCTGTCTGTCGGTGA
*V. albo*-*atrum*	TTTCACGACCGATGAAAGCG	CACATCGGCGAGGATCTGTC
*V. tricopus*	CACCCTCGGGCACACCAATA	TCCGTGGAGGTTGAGCGCTAT

### Naturally infested soil and leaf samples

The species-specific primers, listed in Table 2, were used to analyze the level of natural infestation in 429 soil samples from two Swedish regions with intensive farming. The soil samples had all high clay content, representing characteristics as in soil mixture number four analyzed by Almquist et al. (2016). A study showing that the limit of DNA detection was lowest in samples with high clay content using similar soil handling and DNA extraction methodology as presented here. Among all 429 samples only *V. dahliae* and *V. longisporum* were found, whereas *V. albo*-*atrum, V. tricorpus* were not detected. The fungal DNA content detected in the samples ranged from 5 fg DNA/g soil to 121.62 pg DNA/g soil of *V. longisporum* and from 6 fg DNA/g soil to 137.84 pg DNA/g soil of *V. dahliae*. In the soil samples from southern Sweden, 40% were infested with *V. longisporum* and 7% were infested with *V. dahliae* (Table 3). The distribution was opposite in the western region, where 19% of the soil samples contained *V. dahliae* and only in 9% of the samples *V. longisporum* was identified (Table 3). Both *Verticillium* species were found in two soil samples from southern Sweden, while in the western region each species was only found individually. Most likely this result reflects the random sampling of the soil samples.

**Table 3:** Detection of *Verticillium* species in soil samples derived from different agricultural regions in Sweden

Species	Southern region No. samples	Western region No. samples
*Verticillium longisporum*	120 (40%)	12 (9%)
*Verticillium dahliae*	22 (7%)	25 (19%)
*Verticillium albo*-*atrum*	0	0
*Verticillium tricorpus*	0	0
Total number of tested samples	297	132

The restriction to *V. longisporum* and *V. dahliae* in the Swedish agricultural soils is in line with earlier observations (Johansson et al. 2006). Detection of both species varied from zero to remarkable high levels, which is most likely related to the different crop rotation schemes practiced in these two regions. In the south, combinations of winter oilseed rape, winter wheat, sugar beets and barley are common, whereas in the western regions sugar beets are replaced by oat or field bean. Due to the high market prices, particularly winter oilseed rape cultivation in the southern region has expanded from 24,100 ha in 2006 to 44,700 ha in 2016 (http://www.svenskraps.se). This increased oilseed cultivation likely attributed to the more frequent occurrence of *V. longisporum*. Using the wet sieving method, as high as 164.8 colony-forming units of *V. longisporum* per gram soil were found when sampling 3 weeks post-harvest from a field in the southern region, where winter oilseed rape had been grown (Andersson 2003). This observation highlights the importance of when soil sampling is done.

In addition, we collected soil and leaf samples from 20 sugar beet fields where foliar wilting had been observed. Here, only *V. dahliae* DNA was found in the leaf samples. In the contrary, species distribution in the 20 soil samples was: 20 positive for *V. dahliae* (26.39–103.66 pg DNA/g soil) and 18 positive for *V. longisporum* (0.72–42.37 pg DNA/g soil). These results correspond with earlier experience in Sweden that sugar beets are only infected by *V. dahliae*. It also shows that degradation of *V. longisporum* takes time and it highlights the risk of mechanical spreading long periods after growing host species besides questions concerning crop rotation.

Successful and precise diagnostic analysis of soilborne pathogens depends on many factors, such as the soil sampling procedure and subsequent treatments (drying, milling, sieving, and sample storage), efficient DNA extraction and suitable nucleic acid target sequence to achieve species-specificity. The many sources for non-optimal conditions for this type of molecular diagnosis imply that a careful interpretation of the results is needed. We suggest that soil tests for advisory work on farming practices should be based on different indicative threshold intervals, since the number of cells and thereby DNA in each individual microsclerotium of the two *Verticillium* species studied here varies substantially. It means that any strict correlation between the amount of DNA per gram soil and number of microsclerotia would be misleading. Cells of *V. longisporum* contain approximately twice more DNA than *V. dahliae* (Steventon et al. 2002) which adds to the complexity. A rough estimate based on 50 cells for each individual microsclerotium would equal 1 pg *V. dahliae* DNA alternatively 2 pg *V. longisporum* DNA per gram soil, which could be applied as thumb-rules for calculations based on qPCR-based soil analyses. Similarly, quantities below 100 fg DNA per gram soil should be regarded as trace levels. In light of estimations in other countries particularly on *V. dahliae*, levels above 10 microsclerotia/g soil should lead to serious concerns (Wei et al. 2015). However, economic losses related to different crops cannot be directly compared since there are various degrees of resistance genes introduced in the different crop varieties grown worldwide.

Molecular monitoring of presumed infested soils is becoming an increasingly important management tool and analytic commercial services have been established for example to detect the Brassica clubroot pathogen *P. brassicae* (Wallenhammar et al. 2016). We foresee similar progress comprising other pathogens. In many cases, the same soil sample could be analyzed for the presence of DNA of multiple plant pathogens. Such multi-test would significantly increase the efficiency and at the same time lower the analytic costs for the growers. Proper interpretation of the analytic results and information on the farm history of the soil, the sample is taken from, are keys for implementation of this type of services by advisory groups.
